# Comparison of Tailored Versus Standard Group Cognitive Behavioral Therapy for Shift Worker Insomnia: A Randomized Controlled Trial

**DOI:** 10.3390/clockssleep7020024

**Published:** 2025-05-09

**Authors:** Tanja Grünberger, Christopher Höhn, Manuel Schabus, Belinda Angela Pletzer, Anton-Rupert Laireiter

**Affiliations:** Department of Psychology, Paris-Lodron-University Salzburg, A-5020 Salzburg, Austria; tanja.gruenberger@stud.plus.ac.at (T.G.); christopher.hoehn@plus.ac.at (C.H.); belinda.pletzer@plus.ac.at (B.A.P.); anton.laireiter@plus.ac.at (A.-R.L.)

**Keywords:** insomnia, shift work, tailored therapy, efficacy study, implicit treatment

## Abstract

Shift workers are at increased risk of insomnia. The standard treatment (cognitive behavioral therapy for insomnia) poses significant challenges for this demographic due to irregular work and sleep schedules. New approaches are still considered insufficient due to high attrition or insufficient effectiveness. Our preliminary study identified sleep-relevant state and trait factors (see secondary outcomes) for incorporation into an innovative manual that addresses sleep in an implicit manner. The objective was to reduce the focus on insomnia and to replace regularity-based interventions. With a sample of 55 insomniacs (67.74% male, mean age 41.62 years), standard and customized treatments were compared using pre-treatment, post-treatment, and three-month follow-up measurements (RCT, self-assessment data). Our linear mixed models revealed the main significant effects of the measurement point for the primary (insomnia severity, sleep quality, sleep onset latency, total sleep time, daytime sleepiness) and the secondary outcomes (selection: anxiety/depression, dysfunctional beliefs, arousal, emotional stability, concern). No main effects of the condition or interaction effects were identified. Non-inferiority and equivalence tests demonstrated that the customized treatment is equivalent to standard therapy, which is a favorable outcome in light of the implicit approach. Consequently, this innovative approach warrants further exploration, incorporating the present results.

## 1. Introduction

A considerable proportion of shift workers (26.5% [1]) manifest symptoms consistent with insomnia or shift work disorder (SWD). The consequences of these conditions are well documented and include depression, anxiety, impaired cognitive performance, an increased risk of accidents, and adverse health consequences; see our preliminary study for further details [2]. While current approaches for this population have demonstrated the applicability of adapted forms of cognitive behavioral therapy for insomnia (CBT-I), these approaches are less effective in alleviating sleep problems than in the general population and face high attrition rates due to the challenge of adapting to irregular work and sleep patterns [3,4,5,6].

Given the high prevalence of this condition among shift workers, it can be posited that its relevance goes beyond the individual suffering of those affected (see an overview of the topic in [2]). Consequently, the development of an effective treatment method that is both applicable to and accepted by this target group is of paramount importance. However, to date, no effective solution has been identified.

The prevailing best practice for treating insomnia in the general population is cognitive behavioral therapy for insomnia (CBT-I), a multifaceted approach encompassing sleep restriction, stimulus control, the cognitive restructuring of dysfunctional beliefs about sleep, psychoeducation, sleep hygiene, and relaxation [7]. However, the implementation of CBT-I for shift workers is complex and challenging, as a substantial proportion of these interventions are based on adherence to regularity, which is hardly feasible for individuals who constantly need to re-adapt their rhythmicity due to rotating shifts [8]. Consequently, modified sleep hygiene guidelines and recommendations for shift workers have been devised [9,10,11]. These encompass the recommendation of scheduled naps, exposure to natural light/light therapy in line with working hours, and the use of sunglasses after night shifts. It is noteworthy that these guidelines encompass behaviors and factors that are partially beyond the control of shift workers, such as lighting in the workplace. The recommendation of anchor sleep times, which are based on regularity, has been a subject of extensive prior discussion [2].

However, the prevailing focus of these recommendations for shift workers has been the management of daytime sleepiness and the enhancement of circadian rhythm adaptation, rather than addressing insomnia, despite its treatment’s necessity and the absence of a gold standard. To address this issue, CBT-I has been adapted to meet the specific needs of shift workers to a greater extent [3,4].

Studies examining these adaptations have been met with criticism due to the relatively modest pre-/post changes observed or the ambivalent nature of the results. Often, experimental groups do not differ in a statistically significant way from their respective control groups, whether in terms of sleep hygiene or a low-glycemic diet. Criticisms of these earlier approaches include small sample sizes (e.g., *N* < 20) and methodological weaknesses [4,12,13,14]. A further limitation is the restriction of most studies to a single professional group (e.g., nurses), which hinders the generalizability of the findings; the adapted manuals also feature context-specific content, such as that which addresses nightmares in firefighters [15].

The assessment of the efficacy of adapted CBT-I for shift workers is ambivalent, as would be expected. Standard or slightly adapted CBT-I is effective for alleviating the psychological and emotional problems caused by shift work, but not for sleep problems per se; it is therefore insufficient for shift workers [4,10,16]. Notwithstanding these limitations, a discernible trend emerges: participants diagnosed with primary insomnia demonstrate greater benefits compared to those with shift work disorder.

Previously, insomnia in shift workers was mainly attributed to circadian misalignment [2]. More recent research has shifted its focus onto cognitive factors that are relevant to the development and maintenance of primary insomnia, as proposed in the cognitive insomnia model [17]. Another contemporary approach involves the separation of daytime and nighttime sleep in both treatment and measurement. The findings of these approaches, partially delivered digitally, are encouraging. Specifically, dysfunctional beliefs, particularly within the worry/helplessness subscale, and selective attention to sleep-threatening stimuli decreased, but no difference to good sleepers was found with regard to catastrophizing and difficulty falling asleep [5]. Participants reported that the cognitive elements were the most beneficial aspect of the intervention, while the sleep restriction component was the most challenging [3].

The second approach yielded improvements in insomnia severity and total sleep time for day and night sleep, while reducing cognitive arousal, anxiety, depression, and dysfunctional beliefs about sleep. All participants achieved partial or complete remission. However, the intervention’s complexity and duration (14–22 weeks) seems to be demanding, as it necessitates adherence to a stringent daytime and nighttime sleep schedule, including rising from bed and remaining in darkness when waking up after sleep onset [6].

While recent studies have demonstrated statistically significant improvements, they remain below the threshold of clinical significance and are undetectable using actigraphy data. The high attrition rates, which, in one more recent case, reached 32.6%, along with the apparently lower compliance in this population, are partially explained by scheduling difficulties. Another potential reason is that the available treatments are not yet adequately suitable and sufficient for shift workers [3,4,5,6,10].

This phenomenon has also been observed in purely digital, self-study interventions, where only 69.6% of participants completed all six sessions. While the authors regard this as a high level of engagement in the intervention, this evaluation should be viewed with a degree of skepticism, considering the minimal effort required of the subjects [3].

The remission rates across these studies vary from 39% to 92% (partial or complete remission), depending on the definition of remission utilized [3,4,5,6,10].

A critical evaluation is necessary to ascertain whether the stringent regulations that are enforced throughout the entire 24 h period in certain treatments could further diminish the already low compliance level. Nonetheless, it is also conceivable that these stringent requirements could serve to further accentuate the focus on disturbed sleep. It is well established that this is a significant contributor to the maintenance of sleep disorders per se (e.g., the cognitive insomnia model, the attention–intention pathway [17,18]). These mechanisms, including the attempt to force oneself to fall asleep, should also develop in shift workers if their insomnia becomes chronic.

Consequently, it is advisable to refrain from directly addressing sleep within the therapeutic framework. Furthermore, all elements pertaining to regularity should be eliminated, particularly in this customized therapy. Former recommendations, such as lighting at the workplace or the duration of rest breaks between two shifts, could also trigger reactance and should be avoided, as they cannot be influenced by the employees themselves.

Consequently, it is necessary to consider which elements should be incorporated into the planned treatment.

To this end, our preliminary study examined a number of evidence-based derived factors and variables known to be associated with sleep (for more detailed explanations, see [2]). Our findings identified anxiety, depression, concern, emotional stability, tension, the importance of sleep, attitudes towards shift work, and towards sleep as promising factors to improve the sleep of shift workers.

Based on these results, a novel therapeutic rationale was developed: an insomnia therapy for shift workers, characterized by its implicit approach to sleep.

To ensure that information regarding healthy sleep and shift work was not withheld, this information was made available for self-study prior to the treatment. Throughout the treatment, the emphasis was placed on addressing mood, anxiety, worry/concern, rumination, and problem-solving skills. An online group setting via MS Teams was employed to minimize the participants’ effort while retaining the advantages of the group setting. For a more thorough exposition of the development and content of the manual, please refer to Section 4.

The present study is designed to assess the efficacy and applicability of this novel approach in comparison to the standard treatment. The objective is to answer the following research questions:

(1) Does implicit treatment within this unique approach significantly improve the sleep of shift workers? A review of the extant literature suggests that this approach has not yet been investigated. The present study hypothesizes that a positive manipulation of mood, anxiety, worry/concern, rumination, and problem solving will result in a positive effect on sleep.
(2) Do the explicitly manipulated factors also improve? As they are secondary outcomes, significant improvements in the aforementioned state- and trait-related factors, as well as attitudes and beliefs, are expected as a result of the therapeutic intervention.
(3) Are these improvements stable over a three-month period? It is anticipated that the positive effects will maintain their stability over the course of the follow-up period for all examined characteristics.
(4) How does the efficacy of the new treatment (CBT-I-S) compare to standard CBT-I in a sample of shift workers? The innovative approach of the implicit treatment of sleep warrants an exploratory investigation, which also examines the possibility of inferiority. By comprehensively considering the needs of shift workers and directing the focus of attention away from sleep, superiority over standard therapy would be a desirable result. Given the absence of prior research in this domain, the achievement of equivalent efficacy would be a commendable outcome, providing a solid foundation for future research.

## 2. Results

### 2.1. Flow of Participants and Attrition

**Figure 1:**
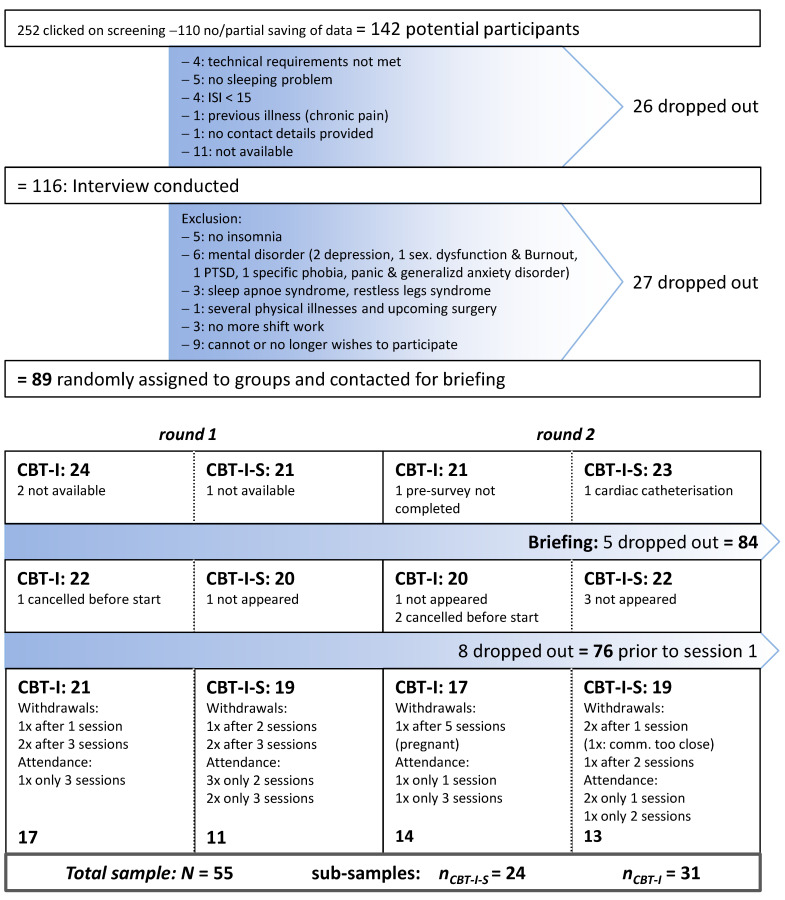
Flow of participants.

Of the 142 subjects who completed the screening, 76 proceeded to the interview and briefing stage (see Figure 1). Despite the meticulous efforts taken during randomization to ensure equivalent group sizes, a discrepancy emerged at the conclusion of the study. Six (CBT-I-S, 15.79%) and four (CBT-I, 10.52%) participants explicitly stated their desire to withdraw from the study. Inadequate attendance is defined as missing more than three of the seven sessions, which applies to eight (CBT-I-S, 21.05%) and three (CBT-I, 7.89%) participants. These figures may include implicit attrition. When the rates of attrition since the commencement of treatment and inadequate attendance are combined, a significant disparity emerges between the two groups: 18.42% for CBT-I and 36.84% for CBT-I-S. Potential explanations for the higher values observed in the CBT-I-S condition are provided in the discussion (Section 3).

No differences were found between dropouts (including inadequate attendance) and completers. Higher values of emotional instability were observed among the dropouts, but this difference lost its statistical significance after α-correction, *t*(74) = −2.78, *p* = 0.133, *Cohen’s d* = −0.71, *CI_95%_* = [–1.23; −0.20].

### 2.2. Recruitment

Participants were recruited throughout Austria and Germany between 21 June 2023 and 1 February 2024. The study was publicized through the distribution of flyers, which included a link to the consent form for participation on the study website. Furthermore, the study was disseminated to approximately 200 companies/institutions with shift work. Additionally, a request was made to 7000 medical professionals (general practitioners, sleep physicians, occupational physicians, psychiatrists) to inform their patients about the study. Finally, participation was featured via social media, regional newspapers and their online offerings, and radio interviews. To address potential impediments, the subjects were provided with an expense allowance of EUR 50. Following a nine-month recruitment period, 142 individuals completed the screening questionnaire, thus bringing the recruitment phase to a conclusion.

Potential participants were provided with comprehensive information about the nature and scope of the study via the website. Following the acceptance of the conditions, the participants were directed to the screening questionnaire. In the event that the inclusion criteria were met, the participants were contacted to arrange a virtual appointment via MS Teams for a diagnostic interview. Following the successful completion of this additional selection procedure, the participants were randomly assigned to either the CBT-I-S or CBT-I condition and subsequently contacted once more for a briefing on the general procedure and scheduling.

### 2.3. Sample Characteristics: Baseline Data

The final sample included 55 participants (67.74% male) with a mean age of 41.62 years (*SD* = 9.30), who had been employed in shifts for 15.26 years on average, *SD* = 10.53. The majority of the participants were Austrian (83.64%). The mean values for the total sample are 38.25 min (*SD* = 22.90) for sleep onset latency and 5.69 h (*SD* = 1.21) for total sleep time. At the initiation of the study, no statistically significant differences were observed between the CBT-I-S and CBT-I groups with respect to their baseline characteristics. A comprehensive account of all distinctive values is presented in Table 1.

### 2.4. Pre-Treatment Improvement

The ISI average score of the total sample at the pre-treatment measurement was 15.00 (*SD* = 3.87), which is precisely the lower limit of clinical relevance of insomnia.

Given that this value also corresponds to the inclusion criterion of ≥15, it was imperative to conduct a thorough examination. A subsequent comparison with the screening data revealed that these values were significantly higher (*M_ISI_* = 23.78, *SD_ISI_* = 3.67), as was intended in the selection of participants. The mean difference between these measurement points was calculated to be *M_diff_* = 8.78, *SD_diff_* = 3.77, and the difference was found to be significant: *t*(54) = 17.30, *p* < 0.001, *d_z_* = 2.33.

The linear mixed model, applied across the ISI values of all four measurement points, yielded a significant main effect of time, *F* = 399.96, *p* = 0.001, η^2^*_partial_* = 0.88, indicating a significant decline in ISI scores over time. Subsequent post hoc tests revealed that all measurement points differed from each other and exceeded the minimal clinically important difference (MCID, ±6), with the exception of the difference between post and follow-up, indicating stability of the improvements.No significant main effect for the condition or a significant interaction effect was identified. The implications of this pre-treatment improvement for the assessment of efficacy are addressed in detail in the discussion.

### 2.5. Primary Outcomes

Given the innovative nature of the newly developed therapy, linear mixed models were employed not only to compare the efficacy of the two conditions, but also to investigate the main effect of the measurement point and interaction effects.

**Figure 2:**
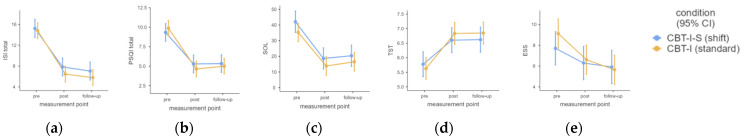
(**a**) Insomnia severity (ISI), (**b**) sleep quality (PSQI total), (**c**) sleep onset latency (SOL), (**d**) total sleep time (TST), (**e**) daytime sleepiness (ESS).

A significant main effect of the measurement point was identified for both conditions, yet no significant main effect of the group or interaction effect was detected (see Table 2 and Figure 2).

### 2.6. Secondary Outcomes

With the exception of a minor significant group effect for sleep hygiene and an equally minor interaction effect for the personality factor tension, only the main effects of the measurement point prove significant for the secondary outcomes. In comparison with the primary outcomes, the effects here are generally less pronounced (Table 3).

### 2.7. Non-Inferiority/Equivalence Tests

**Figure 3:**
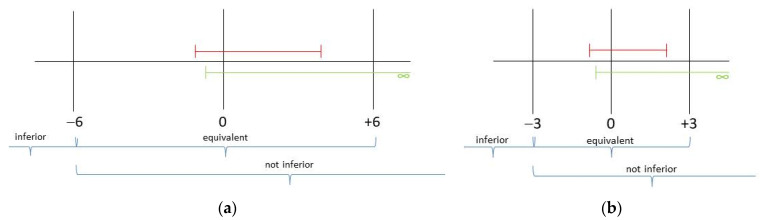
Non-inferiority (green)/equivalence (red) tests of CBT-I-S vs. CBT-I: (**a**) insomnia severity (ISI); (**b**) sleep quality (PSQI).

In light of the findings up to this point, a test for superiority has become obsolete. Table 4 presents the distribution measures for the severity of insomnia and the sleep quality of both conditions, as well as the 95% confidence intervals of the differences. The non-inferiority/equivalence tests are illustrated graphically in Figure 3a,b.

The results demonstrate that both treatments are equivalent in terms of efficacy and that CBT-I-S is not inferior to CBT-I.

### 2.8. Number Needed to Treat (NNT)

Irrespective of the baseline or target value used (see Table 5 and Section 4), the NNT calculations appear at first glance to suggest that CBT-I is slightly superior to CBT-I-S. However, the confidence intervals of the absolute risk reduction (ARR) are very wide and reach into the negative range. Consequently, it is not possible to draw any definitive conclusions regarding the comparative efficacy of the two treatments based on these results (see Table 5).

### 2.9. Perceived Effectiveness

With regard to the subjective evaluation of the treatment’s overall effectiveness, as measured by a categorical question (see Section 4.), no significant difference was found between CBT-I-S (*M* = 3.96, *SD* = 0.91) and CBT-I (*M* = 3.97, *SD* = 1.14); *t*(52.94) = −0.03, *p* = 0.973, n.s. (variances not equal, *F* = 4.18, *p* = 0.046).

The qualitative evaluation of the feedback using thematic analysis also demonstrated parallels between the two conditions, considering the group size and the number of feedback items. Statements classified as “helpful” were more prevalent in the CBT-I-S condition, though improvements in well-being were more often observed in the CBT-I condition. The proportion of feedback responses mentioning an improvement in sleep was similar in both groups.

The terms “well-prepared documents” and “pleasant group atmosphere” were mentioned exclusively by the participants in the CBT-I-S group. Negative feedback following CBT-I-S was observed to be associated, in one instance, with the therapeutic rationale, and, in two instances, with the unchangeability of the underlying causes—specifically, the persistent shift work. Of course, this critique cannot be ascribed to the specific form of treatment. A comparable feedback item was identified in the CBT-I group: namely, that a long-standing problem cannot be resolved within a three-month timeframe. It is noteworthy that the therapy was deemed “unsuitable for shift workers” exclusively by three CBT-I participants (see Table 6).

### 2.10. Treatment Integrity

The treatment was implemented as intended. The trainers of both conditions strictly followed their respective manuals. The subjects also exhibited a high level of adherence to all instructions and completed the prescribed exercises, which were reviewed by the trainers at the commencement of each session.

The issues encountered during implementation were predominantly associated with scheduling or the acceptance of the therapeutic rationale, as detailed in Section 3. It appears improbable that this exerted a considerable influence on the treatment’s integrity, as participants encountering these difficulties often opted to withdraw voluntarily.

## 3. Discussion

The newly developed CBT-I-S manual and the standard CBT-I both resulted in significant enhancements in the primary outcomes sleep onset latency, total sleep time, subjective sleep quality, sleep efficiency, and daytime sleepiness, as well as total sleep quality and insomnia severity. Secondary outcomes, encompassing state and trait factors, also exhibited enhancements across the measurement points, though the effect sizes in these cases are predominantly in the medium range.

The enhancements in sleep quality (PSQI total) and insomnia severity (ISI) surpass the respective minimal clinically important difference in both conditions, thereby indicating clinical relevance. Furthermore, CBT-I-S was found to be non-inferior to CBT-I: both conditions are equivalent. The effectiveness perceived by the participants is comparable in both conditions, while the attrition rate (defined as dropouts and inadequate attendance) is higher in CBT-I-S.

The first hypothesis, which postulated that the implicit therapy approach would enhance the sleep of shift workers, was validated. The second hypothesis posited a positive treatment effect for the state and trait factors, which were addressed explicitly. This was also confirmed, albeit to a lesser extent. The third hypothesis addressed the stability of the results over a period of three months, a premise that was substantiated for both conditions. The expectations regarding the final research question, about the comparative efficacy of the two conditions, were deliberately left open due to the novel nature of the implicit approach. While the hypothesis of treatment equivalence was confirmed, the superiority of CBT-I-S over CBT-I was not substantiated.

The large effects of all the measured variables (with the exception of modest to medium effects pertaining to personality traits) in both conditions across the measurement points indicate the clinical significance of the observed improvements. These values are at least comparable with the more recent studies on shift work (e.g., [6]), which is particularly remarkable and encouraging in view of the implicit approach of CBT-I-S. The statistical equivalence of the approaches is a valuable result. CBT-I has been validated in the general population and, in an adapted form, in shift workers, while the CBT-I-S was here evaluated for the first time. To the best of our knowledge, the interventions integrated here have not yet been used to improve sleep. Consequently, the finding that the two treatments are equally effective is particularly gratifying.

Nevertheless, it is unfortunate that CBT-I-S does not demonstrate superiority over CBT-I. The treatment of sleep disorders in shift workers poses a significant challenge due to the disruption of their circadian rhythm caused by changing work hours and sleep patterns [3,4,5,6]. The implementation of interventions targeting the regularity of the sleep–wake cycle has been found to be challenging, resulting in suboptimal compliance. The novel CBT-I-S approach eliminates these interventions and replaces them as described. This modification was expected to rectify the aforementioned issues; however, this result was not observed. The second approach we used aimed to reduce attention to sleep disturbances. However, the importance of sleep was equally diminished across both conditions. Consequently, this feature does not appear to be a distinctive characteristic of CBT-I-S. In addition, the secondary outcomes, which were explicitly addressed in CBT-I-S but implicitly addressed in CBT-I, showed equivalent improvements in both conditions.

The attrition rates observed in this study are notably high. When contextualized within the broader body of research, these rates do not appear to be exceptional for shift workers (20–58% [4,6]). Given its comprehensive adaptation to the needs of shift workers, it was hypothesized that the attrition rate for CBT-I-S would be lower than for CBT-I. However, the observed rate was twice as high. While the participants’ feedback suggests that CBT-I-S is indeed somewhat easier to implement in everyday life for shift workers, the figures are sobering. A subsequent analysis of dropouts vs. completers failed to identify any discernible factors that could explain the observed differences in attrition rates between the two groups.

In consideration of the observations and feedback provided by the trainers, it is possible to formulate hypotheses regarding the underlying causes. However, it is crucial to note that these assumptions are speculative in nature, as the data were not systematically collected. Inadequate attendance may be primarily attributable to factors such as illness, technical difficulties, or short-term duty substitution, rather than a lack of compliance. However, this explanation would apply to both conditions. Due to the protracted acquisition period, the implementation of the treatment took longer than planned, which led to conflicts with other obligations for the trainers, particularly in the CBT-I-S condition. This impeded their ability to offer dates with the flexibility initially envisioned and agreed upon. Another potential explanation could be that the therapeutic rationale for only implicitly treating sleep has not been sufficiently elaborated. It has been observed that some of the participants exhibited signs of irritation or skepticism towards the efficacy of the method.

The significant pre-treatment decrease in insomnia severity (ISI score) is a concerning observation. As intended, the mean ISI score at screening was in the range of severe insomnia (ISI ≥ 22). By the commencement of treatment, it had decreased to the lower limit of moderate insomnia (15–21). The observed alteration can be attributed to expectation effects. A sizeable body of research has demonstrated a positive correlation between outcome expectations and treatment effects [20]. However, a recent meta-analysis [21] indicates that the effects between pre- and post-treatment measurements are typically modest, which contradicts the magnitude of the observed effect in the present study.

This may be attributed to the observation that shift workers often perceive a lack of available assistance or assume that poor sleep is an unavoidable consequence of their work schedule. This assumption is further substantiated by the fact that a significant proportion of participants expressed their gratitude in their feedback for having the opportunity to participate in this customized treatment program. This may have led to heightened expectations, resulting in an anticipatory improvement in sleep quality.

While this explanation is speculative, the alternative is overly simplistic: that is, exclusively attributing the findings to a “waiting list effect”, which posits that the observed improvement solely results from the passage of time, independent of any therapeutic intervention. However, the absence of a correlation between waiting time and improvement contradicts this hypothesis.

Although all subjects suffered from at least moderate clinically significant insomnia at the time of selection, this condition no longer existed at the start of treatment. This limitation renders the study’s findings less applicable in terms of assessing the efficacy of the treatment for severe insomnia. It is noteworthy that other studies, such as [6], also initiate treatment with a comparable baseline, characterized by a *M_ISI_* below 15. The paucity of studies with higher initial values is notable. Consequently, the lower-than-intended baseline values should be taken into account during interpretation, but they should not be overestimated.

A notable benefit of the novel treatment is its potential to be more accessible for shift workers compared to conventional treatment methods. The interventions employed in the CBT-I-S, which only implicitly address sleep, are evidently suitable for replacing interventions focused on regularity, such as sleep restriction. Notably, the statement “not appropriate for shift workers” was made exclusively by the standard CBT-I group.

This feedback, in conjunction with the overall results, underscores the potential of this approach, while also highlighting the necessity for the further development and refinement of the manual. For instance, the rationale underpinning this approach could be articulated with greater clarity and precision. In addition, a less rigid approach to excluding sleep from therapy may prove beneficial.

### 3.1. Limitations

The limitations of this study are rooted in the utilization of self-assessment data, which is inherently subjective in nature. This subjectivity introduces potential risks of various biases, such as socially desirable response behavior or faking good/bad responses. Additionally, the possibility of a bias in subjective perception without underlying intention cannot be discounted. The incorporation of objective polysomnographic recordings would have enhanced the study’s quality and validity. The decision to forgo laboratory tests was made due to the necessary supra-regional expansion of recruitment and the limited funding for this study.

A notable critique pertains to the cancellation of the planned passive control group, which was due to recruitment challenges. The establishment of a “pseudo-control group” was contemplated, given the availability of screening data for at least insomnia severity. However, this approach was finally not considered feasible due to methodological constraints, as discussed in Section 4. As a consequence, the resulting disadvantages had to be accepted. Utilizing a 3 × 3 design, the significant pre-treatment improvement could possibly have been proven or disproven. In the absence of a control group, the number needed to treat (NNT) could only be calculated by comparing the two conditions. The level of clinical relevance, which the NNT is intended to measure, would have to be calculated against no treatment or a placebo treatment.

The high attrition rate, particularly in the CBT-I-S condition, prompts the question of why this occurred. The ethical guidelines stipulate that participants are permitted to withdraw from the study “without giving a reason”; thus, no data are available on this. It is imperative to ascertain whether the reasons for attrition are actually attributable to the therapeutic modality, which was designed to address the needs of shift workers, or whether there are other reasons.

From a methodological perspective, this high rate of attrition could potentially compromise the study’s validity, but the dropout–completer analysis of the baseline data did not reveal any systematic differences. It is regrettable that there are not enough post-treatment data for an intention-to-treat analysis, meaning that the overall results are based on a per-protocol analysis.

A further methodological critique pertains to the non-normal distribution of certain variables, but it should be noted that this phenomenon is mostly restricted to a single condition. To ensure a standardized and clear evaluation, it was decided that parametric tests should be used for the entire evaluation. We cite [22,23,24] as evidence that *t*-tests are relatively resilient to deviations from a normal distribution. With regard to the linear mixed models and the tests used for equivalence/non-inferiority, we opted for parametric tests due to the absence of non-parametric methods.

It is regrettable that the studies referenced in the introduction and which exhibited considerable promise were not available at the time this study was planned. Had these studies been accessible during the study’s design phase, the overall quality of the study would likely have been enhanced.

### 3.2. Strengths

The present study is outstanding in terms of its innovativeness. To the best of our knowledge, the implicit treatment of sleep disorders has not yet been examined elsewhere. Our approach was developed to redirect the maintenance factor of excessive attention away from sleep disturbance. It also facilitates the replacement of regularity interventions, which are not feasible for shift workers, with interventions for other disorders such as depression and anxiety. The outcomes are highly promising, paving the way for further exploration and the refinement of this novel approach.

Previous studies have frequently been constrained to individual occupational categories. In contrast, this study did not impose any restrictions, allowing for the generalization of its results to different occupational groups. Noteworthy strengths of the study include its high power and supra-regionality, achieved through the online setting. A further noteworthy strength of the study is its concurrent examination of multiple variables, encompassing not only various sleep parameters but also an array of state- and trait-related factors. This multifaceted approach offers a more comprehensive perspective on the underlying mechanisms and implications.

## 4. Methods and Materials

### 4.1. Trial Design

The present study employed a 2 (condition: CBT-I-S vs. CBT-I) ×3 (time: pre-treatment, post-treatment, 3-month follow-up) randomized controlled trial with an ad hoc sample. Due to constraints related to cost and time, it was not feasible to stratify the sample. Nevertheless, the recruitment process was designed with a broad scope to ensure a good representation of gender, age, and profession from German-speaking countries. Recruiting difficulties led to the cancellation of the originally planned third group, designated as a passive control. These challenges also resulted in the extension of the interval between screening and pre-measurement to several months in some cases. This development, however, presented us with an opportunity to compare the ISI values between these two measurement points.

The decision to calculate this analysis separately and not to integrate it as an independent control group was based on several considerations. These included the pronounced inter-individual variation in the waiting time (range 2.22–192.05 days), the lack of correlation between the improvement in insomnia severity and the waiting time, *r*(53) = −0.02, *p* = 0.889, and the fact that only insomnia severity screening data were available; this made a change to a 3 × 3 design appear methodologically incorrect.

### 4.2. Participants

The eligibility criteria for participants encompassed primary insomnia (ISI ≥ 15), engagement in shift work (at least two different shifts or a permanent night shift) for more than 30 h per week, age 18–65 years, and sufficient German language skills. Exclusion criteria included sleep apnea syndrome, restless legs syndrome, chronic pain, acute addiction, affective or anxiety disorder, psychotic, bipolar, and schizo-affective disorder, each in severe form.

All data were collected online. The implementation of screening, pre, post and follow-up measurements was conducted through the administration of online surveys. The eligibility criteria were verified in the form of a diagnostic interview via MS Teams. The implementation of online data collection facilitated the expansion of the recruitment process to encompass the entire German-speaking region. However, this expansion came with the limitation of relying solely on self-reported data, which was considered a disadvantage.

### 4.3. Setting and Conditions of Implementation

The decision was made in favor of an online group setting, as this eliminated travel time for the participants, which could be a decisive factor in view of expected scheduling difficulties. Furthermore, individuals engaged in shift work often experience a heightened sense of social isolation [25], thereby underscoring the potential for constructive group interactions and dialogues. The efficacy of online CBT-I is well documented [26]. Nevertheless, this setting may also entail certain disadvantages. Maintaining complete anonymity is not possible in a group setting; at the very least, faces and first names have to be revealed. In professional contexts where the disclosure of insomnia would have adverse consequences, such as aviation and policing, this could present a significant impediment. In such instances, autonomous implementation, e.g., using mobile applications, might have garnered greater acceptance. It is also conceivable that conducting the group meeting online via MS Teams could have deterred individuals due to potential technological skill gaps.

To ensure comparability, both treatments were designed to be as similar as possible. They were structured into seven sessions, each spanning 90 min. Each session began with questions about participants’ current mood and any challenges encountered with the assigned homework exercises; then, the content of the ongoing session was covered, concluding with the allocation of another homework exercise. Two groups per condition (CBT-I-S vs. CBT-I) commenced in the same week, thus offering an alternative date for each session. Given that only four of the seven sessions were mandatory, the study website provided access to all content in video and text formats to account for missed appointments.

The trainers were advanced Masters students in Psychology from the University of Salzburg, who specialized in Clinical Psychology, completed a clinical internship, and were extensively instructed in the topic and the manual. The implementation of the treatment was overseen by experienced supervisors, who were psychologists and certified cognitive behavioral therapists.

Ten days prior to the first session, the link to the pre-survey was forwarded. Following the completion of the survey, participants were provided with reading material pertaining to CBT-I-S or a sleep diary for CBT-I, as delineated in the respective treatment manuals (Section 4.4). Following the final session, the post-survey link was distributed, accompanied by a request for prompt completion. Three months later, the follow-up survey link was distributed via email.

### 4.4. Treatment Manuals

#### 4.4.1. CBT-I: Standard Manual

It was necessary to adapt a CBT-I manual to align with the online group setting. The interventions were curated in accordance with contemporary recommendations [7] and were derived from several standard manuals (see Table 7). Adjustments were made for the use of CBT-I with shift workers, but these were kept to a minimum. For interventions that are challenging for shift workers, it was recommended to select individually suitable techniques. The unmodified sleep restriction was presented with an emphasis on prioritizing the duration of the sleep window over its timing.

#### 4.4.2. CBT-I-S: Shift-Specific Manual

The treatment should not focus on the issue of disturbed sleep, while ensuring that all pertinent information is disclosed. Consequently, sleep education was provided prior to the treatment for self-study. In the first session, the therapeutic rationale was derived from this information: Address the sleep issues indirectly, thus avoiding the further increase of focus on the problem—a deliberate strategy that is expected to ensure its efficacy. The interventions were primarily derived from cognitive behavioral therapy and positive psychotherapy: namely, treatments for other disorders, e.g., anxiety and depression (see Table 8). No sleep examples were utilized to teach these methods, and the topic of regularity was fully eliminated. The treatment also aimed to mitigate feelings of resignation and helplessness associated with insomnia by enhancing self-efficacy expectations through self-applicable methods.

To address the issue of reduced compliance [10], the consideration of the unique requirements of shift workers within the development of this tailored treatment was emphasized. Moreover, it was necessary to deviate from the standard wording: for example, by referring to the “main sleep phase” rather than to “night sleep”.

### 4.5. Outcome Variables, Measurement Points, and Instruments

Self-report data were collected via online questionnaires (LimeSurvey) at three measurement points: (T1) pre-measurement ten days prior to the first session; (T2) post-measurement following the final session; (T3) follow-up measurement three months after the final session. Online screening (T0) and a diagnostic interview were carried out in advance to verify the eligibility criteria. The outcome variables, questionnaires, and subscales utilized at each measurement point are presented in Table 9.

The left-hand column of Table 9 delineates the outcome variables, which concomitantly signify the measurement content of the instruments employed (right-hand column). All these instruments exhibit at least sufficient reliability and validity; more details on the instruments are provided in the study protocol [46]. The single items developed in-house were used on an item basis.

**Table 9:** Outcomes, instruments, and measurement points.

Content/Outcome Variable	T0: Screening	Interview	T1: Pre	T2: Post	T3: Follow-Up	Instrument/Reference
**Screening and interview: inclusion/exclusion criteria**						
Age, sufficient German language skills, technical requirements, previous illnesses, insomnia (for ≥3 months, ≥3×/week; ISI ≥ 15), weekly working hours ≥ 30, shift work, contact details	×					In-house developed items; ISI (Insomnia Severity Index) [47]
Screening for mental disorders		×				Mini-DIPS (Diagnostisches Interview psychischer Störungen) [48]
Insomnia, no other sleep disordersuch as restless legs syndrome, sleep apnea syndrome		×				SIS-D-5: diagnostic interview, in-house development based onSIS-III-R [49]; DSM-5 [50]; SCID-5-CV [51].
Attitude towards shift work			×			Own item: “Do you like working shifts? Yes, I don’t mind/No, but I have to”
Chronotype			×			rCSM (Reduced Composite Scale of Morningness) [52]
**Demographics**						
Age, gender, federal state, marital status, years of shift work, profession, shift system			×			In-house developed items
**Sleep variables**						
Sleep quality (PSQI total); “subjective sleep quality” (SSQ, comp. 1); sleep-onset latency (SOL, item 2); total sleep time (TST, item 4); sleep efficiency (comp. 4)			×	×	×	PSQI (Pittsburgh Sleep Quality Index) [53]
Insomnia severity (Screening: ≥15)	×		×	×	×	ISI (Insomnia Severity Index) [47]
Daytime sleepiness			×	×	×	ESS (Epworth Sleepiness Scale) [54]
Dysfunctional beliefs about sleep			×	×	×	MZS (Meinungen zum Schlaf Fragebogen) [55]
Importance of sleep (categorical item 1–5)			×	×	×	Own item: “How important is your sleep to you?”
Cognitive and somatic arousal before sleep			×	×	×	PSAS (Pre-Sleep Arousal Scale) [56]
Sleep hygiene			×	×	×	SHI (Sleep Hygiene Index) [57], own translation
**Psychological and personality factors**						
Anxiety, depression, mental well-being			×	×	×	HADS-D (Hospital Anxiety and Depression Scale) [58]
Emotional stability (C), tension (Q4), concern (O)			×	×	×	16 PF-R (16 Personality Factor Test, revised version), [59]
Feedback on therapy: categorical item 1–5; open feedback					×	“Please rate how helpful the training was for you overall:”

Mini-DIPS and SIS-D-5 assessments were conducted via MS Teams; all other instruments were integrated into the online surveys via LimeSurvey.

### 4.6. Power

An a priori power estimation was conducted using G*Power 3.1.9.7 [60]. For the within–between interaction (two conditions, three measurement points), a medium effect size (*f* = 0.25), 1 − β = 0.80, *p* = 0.001 (corrected for multiple outcome variables) and a correlation among repeated measures of 0.5, the required total sample size is *N* = 56. The medium effect size was employed in the power estimation process, as it is regarded as clinically significant [61].

### 4.7. Randomization and Blinding

The randomization and blinding of participants were managed by a blinded study assistant. A computer-generated paired random number series was applied to the subjects in the order of their inclusion. It was not feasible to blind the participants or trainers, as the conditions were clearly discernible due to the content of the treatment. The potential influence of this factor on expectations regarding efficacy could not be prevented or mitigated.

### 4.8. Statistical Analyses

The statistical analyses followed the approach of a completer analysis, as post- and follow-up data were not available for 17 of the 21 attritions (including inadequate attendance).

Potential a priori differences between the conditions were examined using χ^2−^ and *t*-tests. The primary and secondary outcomes were calculated using linear mixed models with two conditions and three measurement points, and the α-values were subsequently corrected [19,62].

Non-inferiority and equivalence tests are necessary to determine whether the CBT-I-S treatment approach was equivalent, superior, or inferior to the established CBT-I treatment [63]. The minimal clinically important difference (MCID) was determined to be the threshold value employed. For the PSQI, this is ±3 [64], and, for the ISI, it is ±6 [65]. The interpretation of the correlation coefficients and effect sizes aligns with the recommendations provided by Cohen [66].

The number needed to treat (NNT) was calculated using different target and baseline values. This was undertaken to provide a comprehensive perspective on the clinical significance of the treatment, despite the pronounced pre-treatment improvement in insomnia severity. The respective instruments’ classifications were utilized for this purpose. ISI: no (0–7), subthreshold (8–14), moderate (15–21), severe (22–28) clinically significant insomnia; PSQI: healthy (0–5), poor sleeper (6–10), chronic insomnia (11–21) [47,53]. For sleep onset latency, a value of at least 30 min was designated as the criterion for integration into the NNT calculation, with a reduction to less than 30 min constituting the target value. Corresponding criteria for other variables, such as the investigated traits, were not available.

Finally, the perceived effectiveness was qualitatively evaluated using a thematic analysis of the participants’ feedback.

All statistical calculations were executed using SPSS 29 [67] or Jamovi 2.3.28.0 [68].

### 4.9. Planned Verification of Treatment Integrity

The trainers were instructed to adhere strictly to the respective manuals. The participants were told that their active cooperation was imperative for their own success and for the study’s outcomes. Given that only four of the seven sessions were mandatory, the study website provided access to all content in video and text formats to account for any potential missed appointments. The trainers were instructed to verify whether absent participants had viewed and understood the content. The trainers were tasked with documenting these aspects of treatment integrity, and the supervisors were responsible for reviewing these records.

## 5. Conclusions

This comparative analysis reveals that the newly developed CBT-I-S and standard CBT-I are equivalent. However, it remains uncertain whether the approach itself is more appropriate for shift workers, given the high attrition rates observed, the reasons for which remain unclear based on the available data. The analysis demonstrates that both conditions exhibit clinically relevant efficacy.

A revision of the CBT-I-S manual is necessary to reflect the experiences and results of this study. Specifically, a more thorough elucidation of the theoretical underpinnings of the implicit therapeutic approach is imperative. Furthermore, the scheduling of appointments should be more flexible: for instance, we could offer one appointment in the morning and one in the afternoon, as explicitly suggested by one participant. For subsequent research, we recommend recording compliance, inquiring about the reasons for attrition, and integrating the novel approaches for shift workers [3,5,6]. Furthermore, the strategy of implicitly addressing sleep in the treatment of insomnia in shift workers merits further investigation, as it has already shown efficacy in its current form and holds considerable promise.

## Figures and Tables

**Table 1:** Sample characteristics and testing of a priori differences.

		CBT-I-S: n = 24	CBT-I: n = 31	Test of a Priori Differences
Age		*M* = 38.88, *SD* = 8.99	*M* = 43.74, *SD* = 9.11	*t*(53) = −1.98, *p* = 0.053, *Cohen’s d* = −0.54, *CI_95%_* = [−1.08; 0.01]
Gender		f = 9 (37.50%), m = 15 (62.50%)	f = 10 (32.26%), m = 21 (67.74%)	χ^2^(1) = 0.16, *p* = 0.685, *Cramer’s V* = 0.055
Pre-existing mental conditions (multiple choice)		1 generalized anxiety disorder, 1 remitted depression, 0 fear of illness, 1 moderate depression, 0 remitted PTSD, 1 remitted anorexia nervosa, 0 restless legs syndrome (independent) 1 mild hypomanic episode	1 generalized anxiety disorder, 3 remitted depression, 1 fear of illness, 0 moderate depression, 2 remitted PTSD, 0 remitted anorexia nervosa, 1 restless legs syndrome (independent), 1 mild hypomanic episode	χ^2^(1) = 1.15, *p* = 0.284, *Cramer’s V* = 0.144
Federal state		12 Salzburg 1 Vienna 2 Lower Austria 2 Upper Austria 2 Styria 1 Tyrol 0 Bavaria 1 Baden–Württemberg 3 North Rhine–Westphalia	14 Salzburg 2 Vienna 1 Lower Austria 6 Upper Austria 3 Styria 0 Tyrol 3 Bavaria 0 Baden–Württemberg 2 North Rhine–Westphalia	χ^2^(13) = 13.96, *p* = 0.377, *Cramer’s V* = 0.354
Marital status		8 single 7 in a partnership 9 married/registered partnership 0 divorced	3 single 8 in a partnership 18 married/registered partnership 2 divorced	χ^2^(3) = 6.56, *p* = 0.088, *Cramer’s V* = 0.504
Waiting period (days)		*M* = 73.49, *SD* = 52.03	*M* = 69.43, *SD* = 38.73	*t*(53) = 0.32, *p* = 0.747
Do you like working shifts?		Yes, I don’t mind: 17 (70.83%); No, but I have to: 7 (29.17%)	Yes, I don’t mind: 17 (54.84%); No, but I have to: 14 (45.16%)	χ^2^(1) = 1.47, *p* = 0.226, *Cramer’s V* = 0.163
Duration of shift work (years)		*M* = 13.61, *SD* = 10.55	*M* = 16.54, *SD* = 10.50	*t*(53) = −1.02, *p* = 0.311, *Cohen’s d* = −0.28, *CI_95%_* = [−0.81; 0.26]
Insomnia severity (ISI, see Section 2.4)		*M* = 15.25, *SD* = 4.32	*M* = 14.81, *SD* = 3.54	*t*(53) = 0.42, *p* = 0.677, *Cohen’s d* = 0.114, *CI_95%_* = [−0.42; 0.65]
Pittsburgh sleep quality index (PSQI)	Subj. sleep quality	*M* = 1.75, *SD* = 0.68	*M* = 1.77, *SD* = 0.50	*t*(53) = −0.15, *p* = 0.879, *Cohen’s d* = −0.04, *CI_95%_* = [−0.57; 0.49]
	Efficiency (%)	*M* = 78.11, *SD* = 9.30	*M* = 74.82, *SD* = 11.57	*t*(53) = 1.14, *p* = 0.261, *Cohen’s d* = 0.31, *CI_95%_* = [−0.23; 0.84]
	Sleep onset latency (min)	*M* = 42.08, *SD* = 26.54	*M* = 35.29, *SD* = 19.58	*t*(53) = 1.09, *p* = 0.279, *Cohen’s d* = 0.30, *CI_95%_* = [−0.24; 0.83]
	Total sleep time (hours)	*M* = 5.78, *SD* = 1.19	*M* = 5.63, *SD* = 1.24	*t*(53) = 0.45, *p* = 0.655, *Cohen’s d* = 0.12, *CI_95%_* = [−0.41; 0.66]
	PSQI total	*M* = 9.33, *SD* = 3.35	*M* = 9.87, *SD* = 2.58	*t*(53) = −0.67, *p* = 0.504, *Cohen’s d* = −0.18, *CI_95%_* = [−0.72; 0.35]
Epworth sleepiness scale (ESS)		*M* = 7.71, *SD* = 4.34	*M* = 9.13, *SD* = 3.96	*t*(53) = −1.27, *p* = 0.211, *Cohen’s d* = −0.34, *CI_95%_* = [−0.88; 0.19]
MZS		*M* = 81.58, *SD* = 20.66	*M* = 76.81, *SD* = 26.23	*t*(53) = 0.73, *p* = 0.467, *Cohen’s d* = 0.20, *CI_95%_* = [−0.34; 0.73]
Pre-sleep arousal scale	Somatic	*M* = 12.00, *SD* = 4.62	*M* = 11.52, *SD* = 3.50	*t*(53) = 0.44, *p* = 0.660, *Cohen’s d* = 0.12, *CI_95%_* = [−0.41; 0.65]
	Cognitive	*M* = 16.88, *SD* = 7.06	*M* = 16.74, *SD* = 6.68	*t*(53) = 0.07, *p* = 0.943, *Cohen’s d* = 0.02, *CI_95%_* = [−0.51; 0.55]
	Total	*M* = 28.88, *SD* = 10.22	*M* = 28.26, *SD* = 8.15	*t*(53) = 0.25, *p* = 0.804, *Cohen’s d* = 0.07, *CI_95%_* = [−0.47; 0.60]
SHI		*M* = 19.33, *SD* = 6,50	*M* = 16.90, *SD* = 5.80	*t*(53) = 1.46, *p* = 0.150, *Cohen’s d* = 0.40, *CI_95%_* = [−0.14; 0.93]
Importance of sleep		*M* = 3.58, *SD* = 0.65	*M* = 3.65, *SD* = 0.62	*t*(53) = −0.75, *p* = 0.455, *Cohen’s d* = −0.21, *CI_95%_* = [−0.74; 0.33]
HADS-D	Anxiety	*M* = 6.25, *SD* = 3.17	*M* = 6.68, *SD* = 3.29	*t*(53) = −0.49, *p* = 0.629, *Cohen’s d* = −0.13, *CI_95%_* = [−0.67; 0.40]
	Depression	*M* = 5.50, *SD* = 3.74	*M* = 5.71, *SD* = 3.55	*t*(53) = −0.21, *p* = 0.833, *Cohen’s d* = −0.06, *CI_95%_* = [−0.59; 0.48]
	Total	*M* = 11.75, *SD* = 6.00	*M* = 12.39, *SD* = 6.09	*t*(53) = −0.39, *p* = 0.700, *Cohen’s d* = −0.11, *CI_95%_* = [−0.64; 0.43]
Personality factors: 16 PF-R	C (emo. stab.)	*M* = 24.83, *SD* = 4.28	*M* = 25.30, *SD* = 3.22	*t*(53) = −0.55, *p* = 0.586, *Cohen’s d* = −0.15, *CI_95%_* = [−0.68; 0.39]
	Q4 (tension)	*M* = 21.63, *SD* = 6.35	*M* = 22.66, *SD* = 5.33	*t*(53) = −0.59, *p* = 0.560, *Cohen’s d* = −0.16, *CI_95%_* = [−0.69; 0.38]
	O (concern)	*M* = 23.71, *SD* = 6.07	*M* = 23.16, *SD* = 6.02	*t*(53) = 0.33, *p* = 0.740, *Cohen’s d* = 0.09, *CI_95%_* = [−0.44; 0.62]
Shift system		2	3	Early and late shift
		9	6	2 shift: day or night shift, 12 h each
		6	8	3 shift: early, late, and night shift
		0	2	3 shift: early, late, and split shift
		0	2	4 shift
		3	1	Always 24 h
		3	1	12 + 24 h mixed
		0	3	Regular hours + at least one 24 h per week or on-call duty
		1	4	Completely irregular
		0	1	Permanent night shift for 12 h
Professions		2	9	Nursing
		1	2	Physician
		2	0	1 midwife/1 medical field
		1	2	Emergency paramedic
		2	0	Fire department
		3	2	Railway
		3	3	Aviation
		1	1	Traffic
		6	5	Police
		1	0	IT support
		1	0	Journalist
		1	7	Production/technology/industry

**Table 2:** Primary outcomes: results of the linear mixed models (α-corrected [19]).

Variable	Measurement Point			Condition			Measurement Point × Condition		
	*F*(2, 106)	*p*	η^2^*_partial_* [*CI_95%_*]	*F*(1, 53)	*p*	η^2^*_partial_*	*F*(2, 106)	*p*	η^2^*_partial_*
Insomnia severity (ISI)	135.02	0.003	0.72 [0.65; 1.00]	1.18	0.414	--	0.39	0.742	--
Sleep quality (PSQI total)	80.24	0.003	0.60 [0.51; 1.00]	0.06	0.843		1.10	0.470	
Sleep onset latency	56.16	0.003	0.51 [0.41; 1.00]	2.00	0.298		0.21	0.843	
Total sleep time	39.04	0.003	0.42 [0.31; 1.00]	0.18	0.742		1.33	0.408	
Daytime sleepiness (ESS)	25.87	0.003	0.33 [0.21; 1.00]	0.25	0.712		2.54	0.174	

**Table 3:** Secondary outcomes: results of the linear mixed models (α-corrected [19]).

Variable	Measurement Point			Condition			Measurement Point × Condition		
	*F*(2, 106)	*p*	η^2^*_partial_* [*CI_95%_*]	*F*(1, 53)	*p*	η^2^*_partial_* [*CI_95%_*]	*F*(2, 106)	*p*	η^2^*_partial_* [*CI_95%_*]
Subjective sleep quality (SSQ)	51.86	0.003	0.49 [0.38; 1.00]	0.30	0.707	-	1.60	0.336	-
Sleep efficiency	40.86	0.003	0.44 [0.32; 1.00]	0.02	0.904		1.79	0.305	
Importance of sleep	7.36	0.003	0.12 [0.03; 1.00]	0.01	0.923		0.71	0.633	
Dysfunctional beliefs about sleep (MZS)	64.12	0.003	0.55 [0.44; 1.00]	1.57	0.341		0.42	0.742	
Somatic pre-sleep arousal (PSAS soma)	6.50	0.006	0.11 [0.03, 1.00]	2.49	0.225		3.56	0.080	
Cognitive pre-sleep arousal (PSAS cogn)	29.69	0.003	0.36 [0.24; 1.00]	2.59	0.219		1.61	0.336	
Total pre-sleep arousal (PSAS total)	22.62	0.003	0.30 [0.18; 1.00]	2.98	0.180		3.19	0.108	
Sleep hygiene (SHI)	21.23	0.003	0.29 [0.17; 1.00]	6.29	0.041	0.11 [0.01; 1.00]	0.60	0.683	
Anxiety (HADS-D anxiety)	20.10	0.003	0.28 [0.16; 1.00]	0.27	0.711		2.55	0.174	
Depression (HADS-D depression)	15.91	0.003	0.23 [0.12; 1.00]	0.59	0.592		3.00	0.125	
Psychological well-being (HADS-D total)	26.25	0.003	0.33 [0.21; 1.00]	0.50	0.633		4.03	0.055	
Emotional stability (16-PF: C)	7.29	0.003	0.12 [0.03; 1.00]	1.11	0.424		1.03	0.492	
Tension (16-PF: Q4)	5.91	0.012	0.10 [0.02, 1.00]	0.35	0.683		5.42	0.017	0.09 [0.02; 1.00]
Concern (16-PF: O)	8.81	0.003	0.14 [0.05; 1.00]	1.23	0.408		2.58	0.174	

**Table 4:** Non-inferiority/equivalence tests of CBT-I-S vs. CBT-I regarding insomnia severity and sleep quality.

Variable	CBT-I-S			CBT-I			Equivalence	Non-Inferiority
	*M*	*SD*	*s* ^2^	*M*	*SD*	*s* ^2^	*CI_95% difference_*	*CI_95% difference_*
Insomnia severity (ISI)	7.83	4.51	20.32	6.45	4.68	21.86	[–1.13; 3.89]	[−0.71; ∞]
Sleep quality (PSQI total)	5.29	2.56	6.56	4.65	2.80	7.84	[−0.83; 2.12]	[−0.58; ∞]

**Table 5:** Absolute risk reduction (ARR); number needed to treat (NNT).

Baseline ISI ≥ 15	Target Score < 15		Target Score < 8	
	Reached	Not Reached	Reached	Not Reached
CBT-I-S	8	5	2	11
CBT-I	14	2	9	7
ARR	25.96%, *CI_95%_* = [−5.05%; 56.98%]		40.87%, *CI_95%_* = [9.63%; 72.10%]	
NNT	3.85 = 4		2.45 = 3	
**Baseline ISI ≥ 8**	**Target Score < 8**			
	**Reached**	**Not reached**		
CBT-I-S	11	12		
CBT-I	21	9		
ARR	22.17%, *CI_95%_* = [−4.01%; 48.36%]			
NNT	4.51 = 5			
**Baseline PSQI >10**	**Target Score < 11**		**Target Score < 6**	
	**Reached**	**Not reached**	**Reached**	**Not reached**
CBT-I-S	11	1	7	5
CBT-I	10	1	7	4
ARR	0.76%, *CI_95%_* = [−22.33%; 23.85%]		5.30%, *CI_95%_* = [−34.52%; 45.13%]	
NNH	132.0 = 132		18.86 = 19	
**Baseline PSQI > 5**	**Target Score <** **6**			
	**Reached**	**Not reached**		
CBT-I-S	14	8		
CBT-I	23	8		
ARR	10.56%, *CI_95%_* = [−14.77%; 35.88%]			
NNT	9.47 = 10			
**Baseline SOL ≥ 30**	**Target Score ≤ 30**			
	**Reached**	**Not reached**		
CBT-I-S	16	1		
CBT-I	20	1		
ARR	1.12%, *CI_95%_* = [−13.30%; 15.54%]			
NNT	89.25 = 90			

**Table 6:** Perceived effectiveness; thematic analysis.

Themes/Categories	CBT-I-S: 13 Open Answers		CBT-I: 17 Open Answers	
	Positive	Negative	Positive	Negative
Thanks	10		10	
Helpful	9	3	8	1
Improvement in sleep	6		9	
Improvement in well-being	2		5	
Unsuitable for shift workers				3
Implementable into everyday life	3	2	3	1
Well-prepared documents	2			
Interesting	1		4	
Pleasant group atmosphere	5		1	
Total training/other	5		5	
scheduling	1			1

Note: multiple categories were mentioned per feedback element.

**Table 7:** CBT-I manual: contents and references.

Sessions	Contents	Quoted from/Based on/Adapted from:
After pre-survey	Sleep diary (to keep until the last session)	[27]
1.	Introduction to the program, sleep education, implementation of relaxation method	[28] (pp. 49–52, 75–78, 95–96); [7,29]
2.	Introduction to sleep restriction, calculation of the first sleep window	[27]; [30] (pp. 87–97)
3.	Deepened sleep restriction, repeat relaxation	[30] (pp. 101–103); [7]
4.	Stimulus control, adaptation of the sleep window, repeat relaxation	[7] (pp. 22–25)
5.	Sleep hygiene, sleep hygiene check; adaptation of the sleep window, repeat relaxation	[28] (pp. 135–141); [7]
6.	Cognitive restructuring of dysfunctional thoughts about sleep	[28] (pp. 174–177)
7.	Sharing experiences, reviewing sleep diaries, relapse prevention, goodbye	[31] (pp. 189–190)

**Table 8:** CBT-I-S manual: contents and references.

Sessions	Contents	Partly In-House Development, Partly Quoted from/Based on/Adapted from:
After pre-survey	Reading material: education on healthy sleep, insomnia, and treatment options	[18,29,30,31,32,33,34,35]
1.	Introduction to therapy Discussion of the reading material Derivation of the therapeutic rationale Effects of attitudes towards shift work	[17,18,29,36,37]
2.	Presentation and discussion of the concept of “shift work tolerance“ Current recommendations for shift workers Positive activities (e.g., social, family, etc.) Daily structure for each shift (early, late, night shift): recognize opportunities “despite shift work”; find an individual relaxation method	[7,9,11,38,39,40]
3.	Central methodologies are employed: systematic problem solving, acceptance, resource orientation.	[41]
4.	(Depressive) rumination: gratitude/happiness diary; grumbling/worrying cessation; relaxation picture	[41,42,43]
5.	Anxiety/concern: decatastrophizing, reality check	[44]
6.	Mood: positive activities, success spoilers, ABC-scheme, cognitive restructuring of dysfunctional (depressive) thoughts	[40,45]
7.	Sharing experiences, emergency kit, relapse prevention, feedback and goodbye	[31]

## Data Availability

Data and manuals will be provided on request (T.G.).

## Abbreviations

The following abbreviations are used in this manuscript:

SWD	Shift work disorder
CBT-I	Cognitive behavioral therapy for insomnia
CBT-I-S	Cognitive behavioral therapy for insomnia in shift workers
TST	Total sleep time
SOL	Sleep onset latency
SSQ	Subjective sleep quality
DS	Daytime sleepiness
ISI	Insomnia severity index
PSQI	Pittsburgh Sleep Quality Index
ESS	Epworth Sleepiness Scale
MZS	Meinungen-zum-Schlaf-Fragebogen (dysfunctional beliefs about sleep)
PSAS	Pre-sleep arousal scale
SHI	Sleep hygiene index
HADS-D	Hospital anxiety and depression scale
16 PF-R	16-personality traits, revised edition
MCID	Minimal clinically important difference
ARR	Absolute risk reduction
NNT	Number needed to treat

## References

[1] Pallesen S., Bjorvatn B., Waage S., Harris A., Sagoe D. (2021). Prevalence of Shift Work Disorder: A Systematic Review and Meta-Analysis. Front. Psychol..

[2] Grünberger T., Höhn C., Schabus M., Laireiter A.-R. (2024). Insomnia in Shift Workers: Which trait and state characteristics could serve as foundation for developing an innovative therapeutic approach?. Preprints.

[3] Ell J., Brückner H.A., Johann A.F., Steinmetz L., Güth L.J., Feige B., Järnefelt H., Vallières A., Frase L., Domschke K. (2024). Digital cognitive behavioural therapy for insomnia reduces insomnia in nurses suffering from shift work disorder: A randomised-controlled pilot trial. J. Sleep Res..

[4] Reynolds A.C., Sweetman A., Crowther M.E., Paterson J.L., Scott H., Lechat B., Wanstall S.E., Brown B.W., Lovato N., Adams R.J. (2023). Is cognitive behavioral therapy for insomnia (CBTi) efficacious for treating insomnia symptoms in shift workers? A systematic review and meta-analysis. Sleep Med. Rev..

[5] Bastille-Denis E., Lemyre A., Pappathomas A., Roy M., Vallières A. (2020). Are cognitive variables that maintain insomnia also involved in shift work disorder?. Sleep Health.

[6] Vallières A., Pappathomas A., Garnier S.B., Mérette C., Carrier J., Paquette T., Bastien C.H. (2024). Behavioural therapy for shift work disorder improves shift workers’ sleep, sleepiness and mental health: A pilot randomised control trial. J. Sleep Res..

[7] Espie C.A., Baglioni C., Espie C.A., Riemann D. (2022). Standard CBT-I protocol for the treatment of insomnia disorder. Cognitive-Behavioural Therapy for Insomnia (CBT-I) Across the Life Span: Guidelines and Clinical Protocols for Health Professionals.

[8] Tout A.F., Tang N.K.Y., Sletten T.L., Toro C.T., Kershaw C., Meyer C., Rajaratnam S.M.W., Moukhtarian T.R. (2024). Current sleep interventions for shift workers: A mini review to shape a new preventative, multicomponent sleep management programme. Front. Sleep.

[9] Järnefelt H., Spiegelhalder K., Baglioni C., Espie C.A., Riemann D. (2022). CBT-I Protocols for Shift Workers and Health Operators. Cognitive-Behavioural Therapy for Insomnia (CBT-I) Across the Life Span: Guidelines and Clinical Protocols for Health Professionals.

[10] Kalkanis A., Demolder S., Papadopoulos D., Testelmans D., Buyse B. (2023). Recovery from shift work. Front. Neurol..

[11] Shriane A.E., Rigney G., Ferguson S.A., Bin Y.S., Vincent G.E. (2023). Healthy sleep practices for shift workers: Consensus sleep hygiene guidelines using a Delphi methodology. Sleep.

[12] Järnefelt H., Lagerstedt R., Kajaste S., Sallinen M., Savolainen A., Hublin C. (2012). Cognitive behavioral therapy for shift workers with chronic insomnia. Sleep Med..

[13] Järnefelt H., Härmä M., Sallinen M., Virkkala J., Paajanen T., Martimo K.-P., Hublin C. (2020). Cognitive behavioural therapy interventions for insomnia among shift workers: RCT in an occupational health setting. Int. Arch. Occup. Environ. Health.

[14] Booker L.A., Sletten T.L., Barnes M., Alvaro P., Collins A., Chai-Coetzer C.L., McMahon M., Lockley S.W., Rajaratnam S.M.W., Howard M.E. (2022). The effectiveness of an individualized sleep and shift work education and coaching program to manage shift work disorder in nurses: A randomized controlled trial. J. Clin. Sleep Med..

[15] Jang E.H., Hong Y., Kim Y., Lee S., Ahn Y., Jeong K.S., Jang T.-W., Lim H., Jung E., Shift Work Disorder Study Group (2020). The development of a sleep intervention for firefighters: The FIT-IN (Firefighter’s Therapy for Insomnia and Nightmares) Study. Int. J. Environ. Res. Public Health.

[16] Takano Y., Ibata R., Machida N., Ubara A., Okajima I. (2023). Effect of cognitive behavioral therapy for insomnia in workers: A systematic review and meta-analysis of randomized controlled trials. Sleep Med. Rev..

[17] Harvey A.G. (2002). A cognitive model of insomnia. Behav. Res. Ther..

[18] Espie C.A., Broomfield N.M., MacMahon K.M., Macphee L.M., Taylor L.M. (2006). The attention-intention-effort pathway in the development of psychophysiologic insomnia: A theoretical review. Sleep Med. Rev..

[19] Benjamini Y., Hochberg Y. (1995). Controlling the False Discovery Rate: A Practical and Powerful Approach to Multiple Testing. J. R. Stat. Soc. Ser. B (Methodol.).

[20] Frank J.D. (1961). Persuasion and Healing: A Comparative Study of Psychotherapy.

[21] Constantino M.J., Vîslă A., Coyne A.E., Boswell J.F. (2018). A meta-analysis of the association between patients’ early treatment outcome expectation and their posttreatment outcomes. Psychotherapy.

[22] Pagano R.R. (2010). Understanding Statistics in the Behavioral Sciences.

[23] Rasch D., Guiard V. (2004). The robustness of parametric statistical methods. Psychol. Sci..

[24] Wilcox R.R. (2012). Introduction to Robust Estimation and Hypothesis Testing.

[25] Cheng P., Drake C.L. (2018). Psychological impact of shift work. Curr. Sleep Med. Rep..

[26] Espie C.A., Kyle S.D., Williams C., Ong J.C., Douglas N.J., Hames P., Brown J.S.L. (2012). A randomized, placebo-controlled trial of online cognitive behavioral therapy for chronic insomnia disorder delivered via an automated media-rich web application. Sleep.

[27] Scharfenstein A., Basler H.-D. (2004). Schlafstörungen. Auf dem Weg zu Einem Besseren Schlaf. Schlaftagebuch.

[28] Binder R., Schöller F., Weeß H.-G. (2020). Therapie-Tools Schlafstörungen.

[29] Crönlein T. (2013). Primäre Insomnie. Ein Gruppentherapieprogramm für den Stationären Bereich.

[30] Müller T., Paterok B. (2010). Schlaftraining. Ein Therapiemanual zur Behandlung von Schlafstörungen.

[31] Scharfenstein A., Basler H.-D. (2004). Schlafstörungen. Auf dem Weg zu Einem Besseren Schlaf. Trainerhandbuch.

[32] American Academy of Sleep Medicine (2014). International Classification of Sleep Disorders (ICSD-3).

[33] Baglioni C., Espie C.A., Riemann D. (2022). Cognitive-Behavioral Therapy for Insomnia (CBT-I) Across the Life Span: Guidelines and Clinical Protocols for Health Professionals.

[34] Pollmächer T., Wetter T.C., Bassetti C.L.A., Högl B., Randerath W., Wiater A. (2020). Handbuch Schlafmedizin.

[35] Kerkhof G.A. (2018). Shift work and sleep disorder comorbidity tend to go hand in hand. Chronobiol. Int..

[36] Åkerstedt T., Sallinen M., Kecklund G. (2022). Shiftworkers’ attitude to their work hours, positive or negative, and why?. Int. Arch. Occup. Environ. Health.

[37] Axelsson J., Åkerstedt T., Kecklund G., Lowden A. (2004). Tolerance to shift work—How does it relate to sleep and wakefulness?. Int. Arch. Occup. Environ. Health.

[38] Reinberg A., Ashkenazi I. (2008). Internal desynchronization of circadian rhythms and tolerance to shift work. Chronobiol. Int..

[39] Saksvik I.B., Bjorvatn B., Hetland H., Sandal G.M., Pallesen S. (2011). Individual differences in tolerance to shift work—A systematic review. Sleep Med. Rev..

[40] Schaub A., Roth E., Goldmann U. (2013). Kognitiv-Psychoedukative Therapie zur Bewältigung von Depression. Ein Therapiemanual.

[41] Teismann T., Hanning S., von Brachel R., Willutzki U. (2012). Kognitive Verhaltenstherapie Depressiven Grübelns.

[42] Feld A., Rudy J.M. (Paris-Lodron-Universität Salzburg, Salzburg, Austria). Coaching der Positiven Psychologie. Manual für Coaches, 2017. Unpublished Manual.

[43] Spiegelhalder K., Backhaus J., Riemann D. (2011). Schlafstörungen.

[44] Becker E., Margraf J. (2002). Generalisierte Angststörung. Ein Therapieprogramm.

[45] Pitschel-Walz G., Bäuml J., Kissling W. (2003). Psychoedukation bei Depressionen. Manual zur Leitung von Patienten- und Angehörigengruppen.

[46] Grünberger T., Höhn C., Schabus M., Laireiter A.-R. (2024). Efficacy study comparing a CBT-I developed for shift workers (CBT-I-S) to standard CBT-I (cognitive behavioural therapy for insomnia) on sleep onset latency, total sleep time, subjective sleep quality, and daytime sleepiness: Study protocol for a parallel group randomized controlled trial with online therapy groups of seven sessions each. Trials.

[47] Gerber M., Lang C., Lemola S., Colledge F., Kalak N., Holsboer-Trachsler E., Pühse U., Brand S. (2016). Validation of the German version of the insomnia severity index in adolescents, young adults and adult workers: Results from three cross-sectional studies. BMC Psychiatry.

[48] Margraf J., Cwik J.C. (2017). Mini-DIPS Open Access: Diagnostisches Kurzinterview bei Psychischen Störungen.

[49] Schramm E., Hohagen F., Graßhoff U., Berger M. (1991). Strukturiertes Interview für Schlafstörungen nach DSM-III-R.

[50] American Psychiatric Association (2013). Diagnostic and Statistical Manual of Mental Disorders.

[51] Beesdo-Baum K., Zaudig M., Wittchen H.-U. (2019). SCID-5-CV. Strukturiertes Klinisches Interview für DSM-5^R^-Störungen—Klinische Version. Deutsche Bearbeitung des Stuctured Clinical Interview for DSM-5^R^ Disorders—Clinician Version von Michael B. First, Jane B. Williams, Rhonda S.

[52] Randler C. (2008). Psychometric properties of the German version of the Composite Scale of Morningness. Biol. Rhythm Res..

[53] Riemann D., Backhaus J. (1996). Behandlung von Schlafstörungen.

[54] Bloch K.E., Schoch O.D., Zhang J.N., Russi E.W. (1999). German version of the Epworth Sleepiness Scale. Respiration.

[55] Weingartz S., Pillmann F. (2009). Meinungen-zum-Schlaf-Fragebogen. Deutsche Version der DBAS-16 zur Erfassung dysfunktionaler Überzeugungen und Einstellungen zum Schlaf. Somnologie.

[56] Gieselmann A., de Jong-Mayer R., Pietrowsky R. (2012). Kognitive und körperliche Erregung in der Phase vor dem Einschlafen. Die deutsche Version der Pre-Sleep Arousal Scale (PSAS). Z. Klin. Psych. Psychoth..

[57] Mastin D.F., Bryson J., Corwyn R. (2006). Assessment of sleep hygiene using the Sleep Hygiene Index. J. Behav. Med..

[58] Herrmann-Lingen C., Buss U., Snaith R.P. (2011). Hospital Anxiety and Depression Scale, Deutsche Version (HADS-D).

[59] Schneewind K.A., Graf J. (1998). Der 16-Persönlichkeits-Faktoren-Test, Revidierte Fassung. 16 PF-R—Deutsche Ausgabe des 16 PF Fifth Edition—Testmanual.

[60] Faul F., Buchner A., Erdfelder E., Lang A.-G., Buchner A. (2007). G*Power3: A flexible statistical power analysis program for the social, behavioral, and biomedical sciences. Behav. Res. Methods.

[61] Moore R.F. (2022). What is an effect size?. Psychiatr. Times.

[62] Hemmerich W. StatistikGuru: Rechner zur Adjustierung des α-Niveaus. https://statistikguru.de/rechner/adjustierung-des-alphaniveaus.html.

[63] Wellek S., Blettner M. (2012). Klinische Studien zum Nachweis von Äquivalenz oder Nichtunterlegenheit. Teil 20 der Serie zur Bewertung wissenschaftlicher Publikationen. [Establishing equivalence or non-inferiority in clinical trials—Part 20 of a series on evaluation of scientific publications]. Dtsch. Arztebl. Int..

[64] Cao S., Xin B., Yu Y., Peng C., Zhu C., Deng M., Gao X., Chu J., Liu T. (2023). Improvement of sleep quality in isolated metastatic patients with spinal cord compression after surgery. World J. Surg. Oncol..

[65] Yang M., Morin C.M., Schaefer K., Wallenstein G.V. (2009). Interpreting score differences in the Insomnia Severity Index: Using health-related outcomes to define the minimally important difference. Curr. Med. Res. Opin..

[66] Cohen J. (1988). Statistical Power Analysis for the Behavioral Sciences.

[67] IBM Corp (2023). IBM SPSS Statistics for Windows.

[68] The Jamovi Project Jamovi. (Version 2.3.28.0). https://www.jamovi.org.

